# Parthenocarpic potential in *Capsicum annuum *L. is enhanced by carpelloid structures and controlled by a single recessive gene

**DOI:** 10.1186/1471-2229-11-143

**Published:** 2011-10-21

**Authors:** Aparna Tiwari, Adam Vivian-Smith, Roeland E Voorrips, Myckel EJ Habets, Lin B Xue, Remko Offringa, Ep Heuvelink

**Affiliations:** 1 Horticultural Supply Chains, Plant Sciences Group, Wageningen University, P.O. Box 630, 6700 AP Wageningen, The Netherlands; 2 Molecular and Developmental Genetics, Institute of Biology, Leiden University, Sylvius Laboratory, Sylviusweg 72, 2333 BE Leiden, The Netherlands; 3 Plant Research International, Plant Sciences Group, Wageningen University and Research Center, P.O. Box 16, 6700 AA Wageningen, The Netherlands; 4 Department of Horticulture, Yangzhou University, Yangzhou, Jiangsu, PR China; 5 Norwegian Forest and Landscape Institute, Høgskoleveien 8, 1431 Ås, Norway

## Abstract

**Background:**

Parthenocarpy is a desirable trait in *Capsicum annuum *production because it improves fruit quality and results in a more regular fruit set. Previously, we identified several *C. annuum *genotypes that already show a certain level of parthenocarpy, and the seedless fruits obtained from these genotypes often contain carpel-like structures. In the *Arabidopsis bel1 *mutant ovule integuments are transformed into carpels, and we therefore carefully studied ovule development in *C. annuum *and correlated aberrant ovule development and carpelloid transformation with parthenocarpic fruit set.

**Results:**

We identified several additional *C. annuum *genotypes with a certain level of parthenocarpy, and confirmed a positive correlation between parthenocarpic potential and the development of carpelloid structures. Investigations into the source of these carpel-like structures showed that while the majority of the ovules in *C. annuum *gynoecia are unitegmic and anatropous, several abnormal ovules were observed, abundant at the top and base of the placenta, with altered integument growth. Abnormal ovule primordia arose from the placenta and most likely transformed into carpelloid structures in analogy to the *Arabidopsis bel1 *mutant. When pollination was present fruit weight was positively correlated with seed number, but in the absence of seeds, fruit weight proportionally increased with the carpelloid mass and number. *Capsicum *genotypes with high parthenocarpic potential always showed stronger carpelloid development. The parthenocarpic potential appeared to be controlled by a single recessive gene, but no variation in coding sequence was observed in a candidate gene *CaARF8*.

**Conclusions:**

Our results suggest that in the absence of fertilization most *C. annuum *genotypes, have parthenocarpic potential and carpelloid growth, which can substitute developing seeds in promoting fruit development.

## Background

Pollination and fertilization are required in most flowering plants to initiate the transition from a fully receptive flower to undergo fruit development. After fertilization the ovules develop into seeds and the surrounding carpels develop into the fruit, while in the absence of fertilization the ovules degenerate and growth of the surrounding carpels remains repressed [1]. The initiation of fruit set can be uncoupled from fertilization, and this results in the development of seedless or parthenocarpic fruits. This can be achieved by ectopic application or artificial overproduction of plant hormones [1], or by mutating or altering the expression of specific genes. In *Arabidopsis*, the *fruit without fertilization *(*fwf*) mutant that develops parthenocarpic fruit [2] has a lesion in the *AUXIN RESPONSIVE FACTOR 8 *(*ARF8*) gene [3]. Expression of an aberrant form of *Arabidopsis ARF8 *also conferred parthenocarpy in Arabidopsis and tomato, indicating ARF8 as an important regulator in the control of fruit set [4]. Mapping of a parthenocarpic QTL in tomato further suggests a role for *ARF8 *in fruit set [5].

Fruit set is normally initiated by two fertilization events occurring in the ovules. Ovules are complex structures found in all seed bearing plants, comprising protective integuments that surround the megagametophyte leaving an opening referred to as the micropyle. When the pollen tube successfully enters the micropyle of the mature ovule, it releases two sperm cells that combine with respectively the egg cell and the central cell. These sites of cell fusion are considered as primary locations from where signalling triggers fruit set [1,6]. After fertilization, the integuments grow and expand to accommodate the developing endosperm and embryo, but they also apparently have a role in coordinating the growth of both fruit and seeds [1]. Various *Arabidopsis *mutants have been identified where ovules show disrupted integument growth, such as *aintegumenta *(*ant*; lacks inner and outer integuments), *aberrant testa shape *(*ats*; contains a single integument), *inner no outer *integument (*ino*; the absence of outer integument growth on the ovule primordium), *short integuments1 *(*sin1*; where both integuments are short), and *bel1 *and *apetala2 *(*ap2) *[7-12]. In the latter two loss-of-function mutants ovule integuments are converted into carpelloid structures [11-13]. Interestingly, two specific mutants have been reported to affect parthenocarpic fruit development of the *Arabidopsis fwf *mutant. Firstly, the *ats-1/kan4-1 *loss-of-function mutation enhances the *fwf *parthenocarpic phenotype, suggesting that modification of the ovule integument structure influences parthenocarpic fruit growth [2]. Secondly, parthenocarpic fruit development was also enhanced in the *bel1-1 fwf-1 *double mutant, and at the same time a higher frequency of carpelloid structures was observed compared to the *bel1-1 *single mutant [14]. This suggests on the one hand that carpelloid structures enhance parthenocarpic fruit development, and on the other hand that the development of carpelloid structures is enhanced in the absence of seed set [14].

Parthenocarpy is a desired trait in *Capsicum annuum *(also known as sweet pepper), as it is expected to minimize yield fluctuations and enhance the total fruit production while providing the inclusion of a quality trait [15]. Research into the developmental and genetic basis for parthenocarpy in *C. annuum *is limited. Several *C. annuum *genotypes have been identified that show tendencies for facultative parthenocarpic fruit development [16]. Seedless fruit from these facultative genotypes display a high frequency of carpelloid structures at low night temperatures [16]. To understand the relationship between parthenocarpic potential and the presence of carpelloid structures, we investigated ovule development and the occurrence of abnormal ovules in *C. annuum *genotypes possessing a range of high (Chinese Line 3), moderate (Bruinsma Wonder) and low (Orlando) potential for parthenocarpic fruit set. Our results show that parthenocarpy in *C. annuum *can promote carpelloid ovule proliferation and that an appropriate genetic background enhances the transformation of ovules which can in turn further stimulate seedless fruit growth. Five selected genotypes that differed most in their parthenocarpic fruit development and carpelloid ovule growth were evaluated to identify a possible correlation between these two traits. Through genetic analysis with crosses between Line 3 and contrasting parents we linked the parthenocarpic potential of this genotype to a single recessive gene. Furthermore sequence analysis showed that the parthenocarpic potential already present in *C. annuum *genotypes is not caused by a mutation in *CaARF8*.

## Results

### Parthenocarpy is widely present in *Capsicum annuum *L. genotypes

To test whether parthenocarpy is widely present in *C. annuum*, twelve genotypes were evaluated for their parthenocarpic potential by emasculating flowers (Table 1). Included in this comparison was Bruinsma Wonder (BW), which has been shown to have moderate levels of parthenocarpy [16]. All genotypes except Parco set seedless fruit after emasculation, indicating a wide occurrence of parthenocarpy in *C. annuum *genotypes (Table 1). Additionally, carpelloid structures were also reported present in most parthenocarpic fruit from the *C. annuum *genotypes previously studied [16], and here we investigate the origin and effect of these structures on fruit initiation.

**Table 1:** Parthenocarpic potential in thirteen genotypes of *Capsicum annuum*

Genotype	Accession number	Number of emasculated flowers	Fruit set (%)
Neusiedler Ideal; Stamm S	CGN21562	66	41
Keystone Resistant Giant	CGN23222	82	39
Yellow Belle	CGN22851	78	38
Sweet boy	CGN23823	58	38
Green King	CGN22122	69	36
Wino Treib OEZ	CGN23270	110	35
Bruinsma Wonder	CGN19226	88	35
Riesen v.Kalifornien	CGN22163	79	34
Florida Resistant Giant	CGN16841	75	32
Emerald Giant	CGN21493	73	32
Spartan Emerald	CGN16846	137	16
California Wonder 300	CGN19189	141	13
Orlando*	De Ruiter Seeds	-	2
Parco	CGN23821	149	0
Lamuyo B*	De Ruiter Seeds	-	0

The accession numbers are from the Center of Genetic Resources, the Netherlands (CGN), The number of emasculated flowers and the percentage of flowers that set into fruit is indicated

*referred from (16)

### Number and weight of carpelloid structures is influenced by genotype

To study whether a positive relation between carpelloid development and parthenocarpy occurs in most of the genotypes of *C. annuum*, we tested five different genotypes, each showing a different potential for parthenocarpic fruit set, at two different temperatures: 20/18°C D/N as a normal temperature and 16/14°C D/N as a low temperature. Previous analysis showed that parthenocarpy is enhanced when plants are grown at low temperature [16]. Pollen viability and pollen germination were significantly reduced at low temperature (P < 0.001) compared to normal temperature (Additional file 1), suggesting that the reduced fertility might enhance the occurrence of observed parthenocarpy. For the non-pollinated category of flowers, pollination was prevented by applying lanolin paste on the stigma of non-emasculated flowers around anthesis. However at normal temperature some flowers were already pollinated before the lanolin application, resulting in seeded fruit (between 1-60 seeds/fruit). At maturity, both seeded and seedless fruits were harvested and the seedless fruits were further characterized into parthenocarpic fruits and knots. Only those seedless fruits that reached at least 50% of the weight of seeded fruits (i.e. only fruits of at least 76 g) were considered as true parthenocarpic fruit, while remaining seedless fruits were considered as "knots", which are characterized as small seedless fruits discarded by industry due to their failure to achieve significant size and colour [16,5]. Taking this criterion into account at normal temperatures Line 3 resulted in 89% seedless fruits (89% parthenocarpic fruits and 0% knots) and 11% seeded fruits while Parco resulted in 78% seedless fruits (56% parthenocarpic fruits and 22% knots) and 22% seeded fruits.

**Figure 1:**
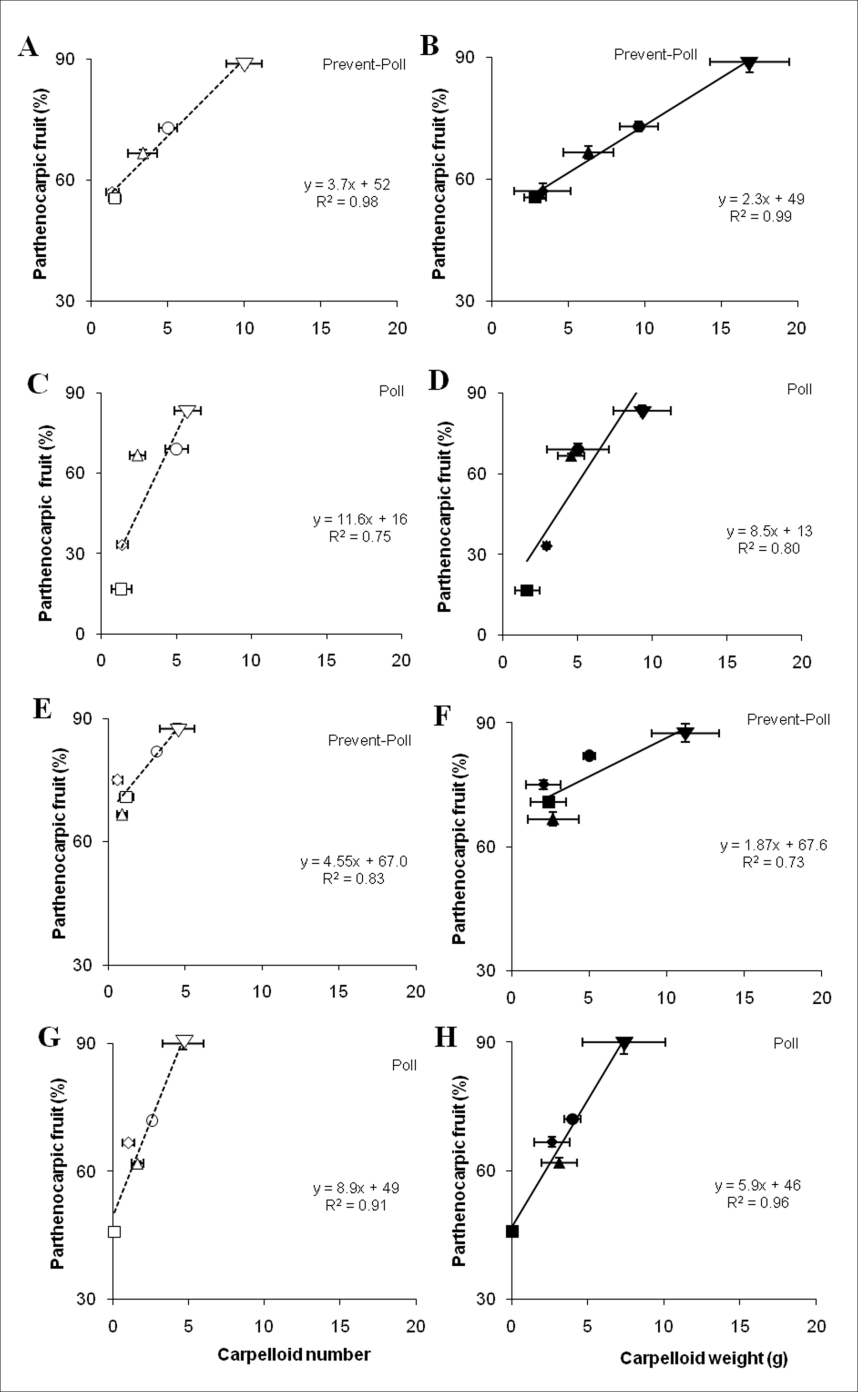
**Genotype-specific evaluation of the percentage of seedless fruits and carpelloids structure (CLS) development**. A-H: Correlation between the percentage of parthenocarpic fruits (only those fruits were counted that reached at least 50% of the weight of seeded fruits) and the mean CLS number (unfilled symbol) and weight (g) (filled symbol) per fruit in the genotypes Parco (***n ***= 18-24) (■, □), California Wonder (***n ***= 18-24) (♦,◊), Riesen v. Californien (***n ***= 18-24) (▲,Δ), Bruinsma Wonder (***n ***= 92-146) (●,o), and Line 3 (***n ***= 18-24) (▼, ∇), at normal 20/18°C D/N (A-D) and low 16/14°C D/N (E-H) temperatures following hand pollination (Poll; C,D,G,H), or prevention of pollination by applying lanolin paste on the stigma at anthesis (Prevent-Poll; A,B,E,F). The regression lines are based on the means of the five ***Capsicum annuum ***genotypes.

At normal temperatures parthenocarpic fruit set and carpelloid growth were clearly genotype dependent (Figure 1), and we observed a strong positive correlation between carpelloid weight and number together with the percentage of parthenocarpic fruit produced. The carpelloid weight was significantly higher in non-pollinated flowers (Figure 1A, B). After preventing pollination, Line 3 showed the highest parthenocarpy (89% of fruits were seedless, excluding knots), and produced the highest number (10 ± 1.16) and weight (17 ± 2.6 g) of carpelloid structures per fruit. In contrast, Parco showed lowest parthenocarpy (56%) with the lowest number and weight of carpelloid structures per fruit (1.6 ± 0.37 and 2.8 ± 0.7 g, respectively; Figure 1A-B). Even after hand pollination, a positive relationship between the number and mass of carpelloid structures and the level of seedlessness was observed (Figure 1C-D).

Evaluation of the same five genotypes at the low temperature regime showed increased parthenocarpy but decreased carpelloid growth though the correlation between parthenocarpy and carpelloid structures remained present (Figure 1E-H). Furthermore, at low temperatures (16/14°C D/N) lanolin application promoted the production of seedless fruits in each cultivar. This resulted for Line 3 in 88% parthenocarpic fruits and 12% knots while Parco had 71% parthenocarpic fruits and 29% knots. Again Line 3 showed the highest parthenocarpy with the highest number (4 ± 1.1) and weight (11 ± 2.2 g) of carpelloid structures, in contrast to Parco where the lowest level of parthenocarpy was observed concomitantly together with a low number (1 ± 0.44) and weight (2 ± 1.15 g) of carpelloid structures (Figure 1E-F). A positive correlation between the presence of naturally occurring parthenocarpic fruit and carpelloid structures was also observed in pollinated flowers (Figure 1G-H). In conclusion, under different temperature conditions and after different treatments (i.e. pollination and where pollination was prevented), a positive correlation was observed between percentages of parthenocarpic fruits and the final number and weight of carpelloid structures.

### The occurrence of abnormal ovule development in *C. annuum*

**Figure 2:**
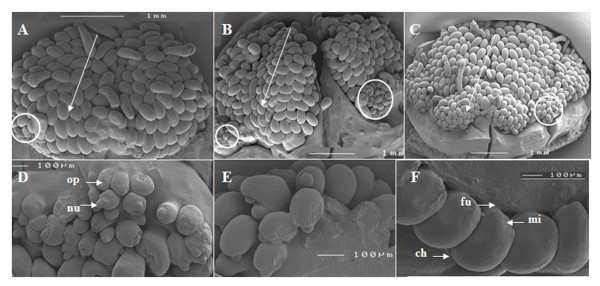
**Cryo-scanning electron microscopy images of ovule development in *Capsicum annuum***. A-C, Comparison of genotypes Orlando (A), Bruinsma Wonder (B), and Line 3 (C) grown at 20/18°C D/N. Gradient of ovule development from top to bottom (arrow head; small circle: undeveloped ovules) Bar = 1 mm. D,E, Ovule primordia (op) initiated from the placenta (arrows), and differentiated in nucellus (nu), chalaza (ch) and funiculus (fu), integument development (E) and development of the micropyle (F). F, Single integument (unitegmic) ovules with micropylar end (mi) situated near the base of the funiculus and oriented towards the placenta (anatropous). Bar = 100 μm.

To study the basis of both parthenocarpic potential and carpelloid proliferation we used scanning electron microscopy to assess deviations in ovule development in specific *Capsicum *genotypes. *C. annuum *has an axillar placenta, where ovules develop in a gradient from top to bottom as shown in genotype Orlando (OR), BW, and Line 3 (Figure 2A-C). Normally the ovule primordium initiates as a protrusion from the placental tissue, and this differentiates into three main proximal-distal elements, respectively known as the funiculus, the chalaza and the distally-located nucellus. The funiculus is comprised of a stalk-like structure and often contains vascular tissues that connect the ovule to the placenta. The chalaza in *Capsicum *is characterized by the presence of a single integument, indicating that the ovule is unitegmic in nature. This integument gradually grows to cover the nucellus leaving a micropylar opening. Typical for an anatropous ovule, at anthesis the micropylar end is oriented towards the placenta (Figure 2D-F).

**Figure 3:**
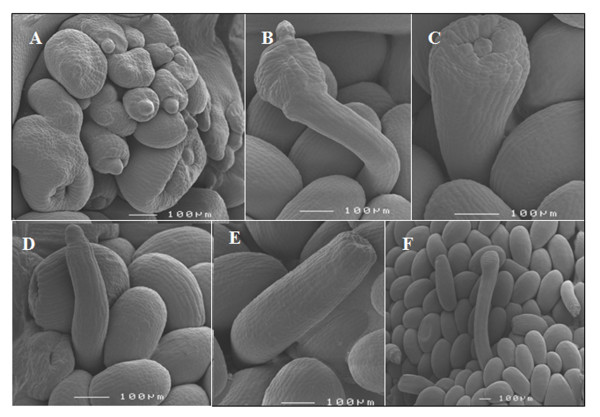
**Cryo-scanning electron microscopy images showing abnormal ovule development in *Capsicum annuum *genotypes**. A-F, Abnormalities detected in the three genotypes were excessive integument growth (A), or carpelloid proliferation of integuments and or the incomplete coverage of the nucellus (B), integuments failing to cover the nucellus (C). In some, ovule structures the integuments partially recurved (D) or were absent (E). Some ovule primordia lacked chalaza and nucellus specification (F). Bar = 100 μm. Genotypes Orlando, Bruinsma Wonder and Line 3 grown at 20/18°C D/N were used for observation.

*Capsicum *genotypes OR, BW, and Line 3 each contained abnormal ovules, which were most abundant at the top and base of the placenta. Ovule abnormalities were most often detected after the integument growth had been initiated and various types of integument abnormalities were observed. For example integument development expanded abnormally across the ovule primordia or proximo-distally to form carpelloid structures (Figure 3A, B). In some cases the funiculus failed to cease growth at the normal length and the nucellus expanded, forming excessively long ovules in which the integument failed to cover the nucellus (Figure 3B). In other cases the integument failed to cover the nucellus, as the integument-like structure did not proliferate from the distal but rather from the more proximal end (Figure 3C). Ovule primordia were also observed to be transformed into amorphic or staminoid tissues (Figure 3D). Others lost the normal anatropous development and took on a "hairdryer" phenotype, reminiscent of the *superman *phenotype [17] (Figure 3E) or only differentiated into a funiculus lacking distal elements (Figure 3F).

### Abnormal ovule development correlates with reduced seed set and enhanced development of carpelloid structures

**Figure 4:**
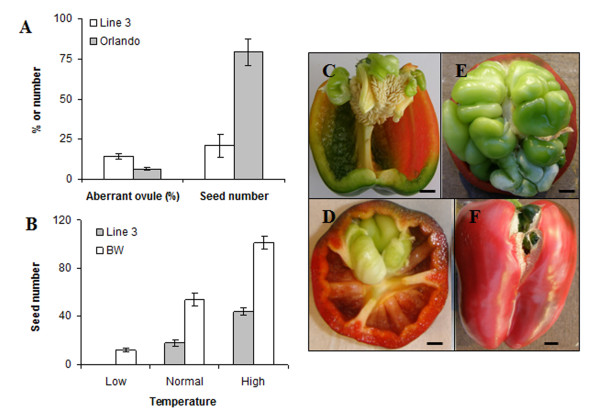
**Genotype-dependent seed set and aberrant ovule frequencies, and phenotypes of carpelloid structures in *Capsicum annuum ***. A: percentage of aberrant ovules (6 gynoecia per genotype), and average seed number in genotypes Line 3 (***n ***= 5) and Orlando (***n ***= 55), B: Average seed number in genotype Line 3 (***n***= 18 at low and normal, and 269 at high temperature) and Bruinsma Wonder (BW, ***n ***= 146 at low, 92 at normal and 167 at high temperature) grown at day/night temperature of 16/14°C (low), 20/18°C (medium) and 22/20°C (high). Data are expressed as mean ± standard error of the mean. C-F: structure and position of CLS in fruits. CLS developing at the basal placental position in seeded fruits (C), or in seedless fruits showing minor (D) or strong (E) CLS growth, or extreme CLS growth resulting in a split at the fruit valve (F). Scale bars: 1 cm.

To test the effect of aberrant ovule development on seed set and carpelloid growth, we quantified the number of aberrant ovules in genotypes Line 3 and OR by evaluating six gynoecia per genotype and 20-30 ovules per gynoecium, and we quantified the seed number by evaluating fruits in Line 3 (*n *= 5) and OR (*n *= 55). The percentage of aberrant ovules was significantly higher in Line 3 compared to OR (14% versus 6%, *P *= 0.001), while the number of seeds was lower in Line 3 compared to OR (21 versus 79, *P *= 0.040) (Figure 4A). Carpelloid growth was already observed within a week after anthesis in Line 3, and after 2 weeks in OR, suggesting early development in Line 3.

To evaluate a possible role of reduced female fertility as a cause of reduced seed set in Line 3, we quantified the number of seeds in Line 3 and BW at low, normal and high night temperature. Pollination was done by vibrating the main shoot two times per week. Previously, 20°C was reported as an optimum temperature for flowering and fruit set in *C. annuum*, and a temperature below 16°C was reported to increase the percentage of seedless fruit [18,19]. Therefore we contrasted 20/18°C D/N with 16/14°C D/N as a low temperature and 24/22°C as a high temperature. The number of seeds was always lower in Line 3 compared to BW at low (0 versus 34 ± 1.5), normal (18 ± 2.8 versus 54 ± 5.1) and high temperature (44 ± 2.8 versus 101 ± 5.5) (Figure 4B). Thus, in Line 3 the high number of abnormal ovules correlated with a precocious occurrence of carpelloid structures and lowered seed set, suggesting that the ovule semi-sterility might also be in part related to the parthenocarpic potential in Line 3.

In all three tested genotypes (OR, BW and Line 3), carpelloid structures were observed as internal green abnormal structures arising from the placenta. The carpelloid structures often had an extensive growth from the placenta (Figure 4C-F). They varied in size from small to large, and in appearance, as mildly (Figure 4D) to severely deformed (Figure 4E). Most of the time the carpelloid structures remained green even after ripening of the fruits and stayed firmly attached to the placenta. Only occasionally, red coloured carpelloid structures were observed in a ripe fruit. The size and weight of carpelloid structures increased with the age of the fruit and for some fruits the carpel margin boundaries were split as carpelloid structures continued to grow to the outside of the fruit (Figure 4F).

### Correlation between carpelloid structures and fruit size in phytohormone-induced parthenocarpy

We used the genotype BW that has moderate parthenocarpic potential [16], to test and observe the relationship between carpelloid growth and seed set, and the effect of phytohormone application on carpelloid proliferation. To obtained seedless fruits, flowers were emasculated prior to anthesis and lanolin paste was applied at anthesis. Emasculated flowers treated with or without hormones (NAA, GA_3_), resulted in only seedless fruits. Emasculation alone resulted in low fruit set (25%). Hormone application on emasculated flowers improved fruit set (30% for NAA, 38% for GA_3_) compared to fruit set obtained after natural pollination (28%). However, the final fruit fresh yield (excluding knots) was higher in seeded fruits (9.7 kg/m^2^) compared to seedless fruits (NAA; 6.9 kg/m^2^, GA_3_; 6.2 kg/m^2^, Em; 4.3 kg/m^2^).

**Figure 5:**
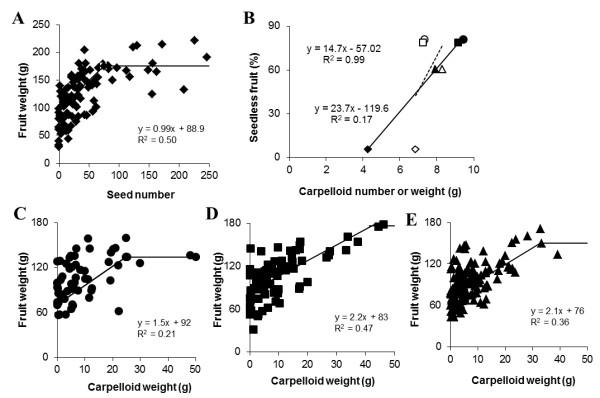
**Relationship between fruit weight, seed set and carpelloid development in *Capsicum annuum *genotype 'Bruinsma Wonder'**. A: A positive correlation between fruit weight (in grams) and seed number up to about 100 seeds (***n ***= 101). B: positive correlation between percentage of seedless fruit and CLS weight (closed symbols, solid line, R**^2 ^**= 0.99) but not with CLS number (open symbols, dashed line, R**^2 ^**= 0.17). Fruits obtained from untreated flowers (♦, ◊), emasculated flowers (●, o), or emasculated flowers that were treated with NAA (■, □) or GA**_3 _**(▲, Δ). C-E: Positive correlation between fruit weight (excluding CLS weight) and CLS weight in fruits obtained from C: emasculated (***n ***= 57), D: NAA treated (***n ***= 84), or E: GA**_3 _**treated (***n ***= 139) flowers. Only fruits of at least 76 g were considered as parthenocarpic and were used for our analysis.

In seeded fruits a positive correlation was observed between fruit fresh weight and seed number up to about 100 seeds (Figure 5A). For seedless fruits, only those fruits that reached at least 50% of the weight of seeded fruits were considered as parthenocarpic fruit and were used in the analysis. More than 90% of both seeded and seedless fruits showed carpelloid structures on their placenta. The average number of carpelloid structures did not differ between seeded and seedless fruits (*P *= 0.382), but the average weight of carpelloid structures was significantly higher in parthenocarpic fruits (*P *< 0.001) (Figure 5B). However, external application of hormones did not influence carpelloid proliferation in either mean number or mass compared to emasculation alone (number of carpelloid structures for Em 7.3 ± 0.7; Em+GA_3_, 8.3 ± 0.4; Em+NAA, 7.2 ± 0.6; weight in Em 9.4 ± 1.0 g; Em+GA_3 _7.9 ± 0.6 g; Em+NAA, 9.2 ± 0.8 g). Thereforeeven with various treatments a positive correlation between seedless fruit (%) and carpelloid weight was observed (Figure 5B). Furthermore, it was observed that seedless fruit weight, excluding carpelloid structures, increased proportionally with the internal carpelloid mass (Figure 5C-E), suggesting a strong synergistic effect between the presence of carpelloid structures and seedless fruit growth.

### Inheritance of parthenocarpy and the relationship with CLS

To study the genetic basis and inheritance of the parthenocarpic potential in *C. annuum*, the parthenocarpic genotype Line 3 was crossed with the non-parthenocarpic parents Lamuyo B, OR F_2_#1 (a male sterile plant selected from an F_2 _population) and Parco. Since Line 3 is a small fruited genotype (Additional file 2; with an average fruit weight of 121 g) and Lamuyo B is a large fruited genotype (average weight of 208 g for seeded fruit; [16], fruit size traits segregated independently upon crossing. This precluded fruit size as the sole criterion to distinguish fruit from knots as discussed earlier. Instead, we took the appearance of fruit as the criterion to distinguish true seedless fruit of small size (shiny appearance, additional file 2 C-E) from knots (dull appearance, additional file 2 D-H). In the F_2 _analysis, a plant was considered parthenocarpic when emasculated flowers all produced seedless fruits showing a shiny appearance. In all three F_2 _populations parthenocarpic plants were observed in 1:3 ratios. Furthermore when the F_1 _of Line 3 × Lamuyo B was backcrossed with Line 3, parthenocarpy was observed in a 1:1 ratio. These data support the hypothesis that parthenocarpy present in Line 3 is controlled by a single recessive gene (Table 2). The same F_2 _plants were evaluated for the occurrence of carpelloid structures. We used two different criteria to distinguish carpelloid from non-carpelloid plants; (i) a less stringent one where plants were scored as having the carpelloid trait if all the true seedless fruits contained at least one carpelloid structure and plants with no seedless fruits were excluded from the analysis and (ii) a more stringent one by which plants were scored as having the carpelloid trait if more than 75% of all the true seedless fruits contained at least one carpelloid structure and plants with less than two seedless fruits were excluded from the analysis. However, taking either criterion into consideration, no mono- or digenic-models could explain with any level of significance the observed carpelloid/non-carpelloid segregation pattern.

**Table 2:** Analysis of segregating population for parthenocarpic fruit set

Crossing	Generation	Expected ratio	Total	Parthenocarpic			
				
				O	E	X^2^	*P*
Line 3 × Lamuyo B	F2	1:3	42	10	10.5	0.03	0.86
	F1 × Line 3	1:1	41	20	20.5	0.02	0.88
Line 3 × OR F_2_#1	F2	1:3	62	17	15.5	0.19	0.66
Line 3 × Parco	F2	1:3	24	5	6.0	0.22	0.64

F_2 _population analysis for parthenocarpy in crosses of Line 3 × Lamuyo B, Line 3 × OR F_2_#1 and Line 3 × Parco, tested by chi-square distribution assuming monogenic recessive inheritance. (O: observed, E: expected, P: probability)

Ninety-four percent of the fruits of Line 3 and 40% of OR F_2_#1 fruits contained carpelloid structures. Both the average number (*P *< 0.001) and the weight (*P *= 0.011) of carpelloid structures per seedless fruit was higher in Line 3 than in OR F_2_#1 at 21/19°C D/N temperature. This agrees with the results described above that the genotypes with a higher potential for parthenocarpy always produced more carpelloid structures.

### Parthenocarpic potential in *C. annuum *is not caused by a mutation in *CaARF8*

Similar to tomato and *Arabidopsis*, a mutation in the *ARF8 *gene might lead to the parthenocarpic phenotype in Line 3. Sequence analysis was performed for a contiguous section of 7508 bp for *CaARF8 *(including 1816 bp of the promoter region plus part of the 3'UTR) in Line 3, BW and OR (Additional file 3). Differences in the sequence were not observed between any of the three genotypes (Addition file 3), indicating that the differences in parthenocarpy are not caused by mutations in the *CaARF8 *gene.

## Discussion

### Most *C. annuum *genotypes have parthenocarpic potential

As an initial step in our attempt to characterize parthenocarpy in *C. annuum*, we tested several genotypes for their potential to set seedless fruits following emasculation. In line with our previous findings [16], most *C. annuum *genotypes developed seedless fruits following emasculation (Table 1), suggesting that some degree of intrinsic parthenocarpy is already present in these genotypes. Genetic variation for the strength of parthenocarpic fruit development was observed (Figure 1), which may occur due to genotypic differences in endogenous auxin and/or gibberellin content in the ovaries or placenta. Genotypes with high potential for parthenocarpy could contain higher levels of hormones compared to those with a lower potential [20]. Intriguingly, however, we also observed that the genotype with the highest parthenocarpic potential (i.e. Line 3) showed reduced female fertility and seed set, and developed significantly more aberrant ovules as compared to the genotype for which no seedless fruit development was observed (OR). Pollination at higher temperatures did not lead to complete seed set in Line 3 whereas it did in BW, supporting the hypothesis that reduced female fertility is associated with enhanced parthenocarpy in Line 3. This hypothesis is corroborated by our previous observation that the expression of parthenocarpy was most prominent in Line 3 (100%) and Lamuyo B (70%) at low night temperature, which leads to further reductions in male fertility (Additional file 1), while this was reduced in Line 3 (73%) and not detectable in Lamuyo B (0%) at normal night temperature [16]. Reduced fertility from aberrant ovules and aberrant anther development is an associated or perhaps even a causal developmental phenotype leading to parthenocarpy in the tomato *pat *mutant (*pat *allele) [21]. Precocious carpelloid growth was observed in Line 3 compared to OR, suggesting that Line 3 contains traits leading to precocious parthenocarpy and or carpelloid transformation well before fertilization. Likewise it has been reported that parthenocarpic fruit development is characterized by autonomous and precocious onset of ovary development in tomato and Arabidopsis [2,22].

### Number and mass of carpelloid structures is influenced by genotype

Carpelloid development was observed in all *C. annuum *genotypes tested, which is in agreement with Lippert [23] who reported that carpelloid structures are present in a wide range of *Capsicum *varieties, but are most commonly observed proliferating in accessions with the bell or blocky type of fruit which have an axial type placenta. Here we show that the resulting number and weight of carpelloid structures was genotype dependent (Figure 1A-H) and that carpelloid development was observed in genotypes possessing a high potential for parthenocarpy. This suggests both traits synergistically interact with one another, or that parthenocarpy promotes proliferation of aberrant ovule primordia. Interestingly, the severity of carpelloid structure is reported to be ecotype dependent also for the *Arabidopsis bel1 *mutant [11]. Though the identity of the ecotype enhancer is unknown, several other genetic loci have co-occurring carpelloid-parthenocarpy proliferation. These are the *Arabidopsis knuckles *mutant which is defective in the *MAC12.2 *gene and the tomato mutant *tm29*, where the down regulation of *TM29 *(*SEPALLATA *homolog) transcription factor results in similar synergistic development of carpelloid tissue proliferation and parthenocarpy [24,25]. This possibly points to a consistent regulatory link between both traits [25].

In most flowering plants, flowers consist of sepals (first whorl), petals (second whorl), stamens (third whorl), and pistils (fourth whorl) [26]. In the Arabidopsis *fwf-1/arf8-4 *mutant, the third whorl organs have an inhibitory effect on parthenocarpic silique development, [2]. In the male sterile *pop1/cer6-1 *background, the *fwf-1/arf8-4 *parthenocarpy mutation only induces strong silique growth when the stamens are removed. The requirement of emasculation is negated when the *pop1/cer6-1 - fwf-1/arf8-4 *double mutant is combined with the *ats-1/kan4-1 *mutant, which has a lesion in ovule integument development [2]. This suggests that the inhibitory signal derived from the stamens, in the third whorl, acts through the ovule integument (fourth whorl) to retard parthenocarpic silique development in *fwf-1/arf8-4 *[2]. In *C. annuum*, we observed parthenocarpic fruit set was enhanced by carpelloid structures. Assuming that carpelloid structures are a form of homeotically converted aberrant ovules, their growth could be governed partially by third whorl identity regulators, but the functions regulating fruit set, either through an independent or a shared pathway, need to be further examined.

### Inheritance of parthenocarpy and relation between parthenocarpy and carpelloid structures

The expression of parthenocarpy in the *C. annuum *genotype Line 3 is facultative, producing seeded and or seedless fruits depending on growth conditions but some semi-sterility is present. We studied the inheritance of parthenocarpy in Line 3 at normal temperatures by using emasculation, and found that the parthenocarpic potential in Line 3 is linked to a single recessive gene (Table 2). Recessive mutations inducing facultative parthenocarpy have been reported before in tomato, citrus and Arabidopsis [2,5,27]. Mutations in Arabidopsis *ARF8 *can provide parthenocarpy, but it can also be obtained when defective forms of *ARF8 *are expressed in Arabidopsis and tomato [4]. In the *C. annuum *cultivars tested the *CaARF8 *sequences were indifferent, excluding that the occurrence of parthenocarpy is caused by a mutation in the coding region of this gene.

In our F_2 _analysis no simple inheritance pattern was observed for carpelloid growth and no clear genetic relationship could be established between the presence of carpelloid structures and parthenocarpy. Perhaps a reason for this is that all parental genotypes used in the three crosses showed some degree of carpelloid transformation (92% fruits with carpelloid structures in Line 3, 56% in Lamuyo B, 46% in OR [16]. The ubiquitous nature of carpelloid structures, but synergistic interaction with parthenocarpy, suggests a non-mendelian inheritance. In order to study the inheritance of this trait, a parental genotype completely devoid of carpelloid growth would be needed. Additionally the strong association between parthenocarpy and carpelloid structures indicates that breeding for high parthenocarpic potential in the absence of carpelloid development will be an important challenge for breeders to overcome.

### Abnormal ovule development and reduced seed set, enhanced carpelloid development and parthenocarpic fruit size

*C. annuum *has an axillar placenta where ovules develop in a gradient from top to bottom. The majority of the ovules are anatropous and unitegmic, as is characteristic for the *Solanaceae *family [28]. Deviations in normal ovule development were observed mainly at the top and base of the placenta (Figure 2), which might be due to abnormal integument growth. A similar pattern of deviations was reported in *Arabidopsis *and petunia where abnormal integument growth resulted in an abnormal ovule mainly at the top and the base of the placenta [29,30] and Cochran [31] showed that carpelloid structures histologically resemble carpel tissue. Stunted integuments in some *Solanaceae *may have a genetic basis since Angenent and co-workers [30] suggested that reduced resource availability may lead to aberrant ovule growth in petunia. The genotype Line 3 contained a high fraction of aberrant ovules and also contained high carpelloid growth compared to OR. Although our data can not exclude that carpelloid structures arise *de novo *directly from the placenta, it is likely that the majority result from homeotic ovule primordia conversions.

An inverse relation between the percentage of aberrant ovules and seed number was observed when comparing the genotypes Line 3 and OR (Figure 4A), suggesting semi-sterility is present in Line 3. This might explain why pollination even at normal and high night temperature did not improve the seed set in Line 3 compared to BW (Figure 4B). Moreover it may be the reason why the parthenocarpic potential is higher in Line 3 as compared to other genotypes. The reduced fertility might allow a window of opportunity for increased expression of parthenocarpic potential.

In general, fruit weight was positively correlated with seed number and in the absence of seeds fruit weight proportionally increased with the carpelloid mass (Figure 5A-F). This suggests that carpelloid growth could substitute for growth signals that normally occur only after pollination and fertilization, mimicking the role of developing seeds. In the absence of fertilization, carpelloid structures can acquire available assimilates and grow prominently, but seeds could however compete better for the assimilates explaining the inverse relationship (Figure 4A)

## Conclusions

**Figure 6:**
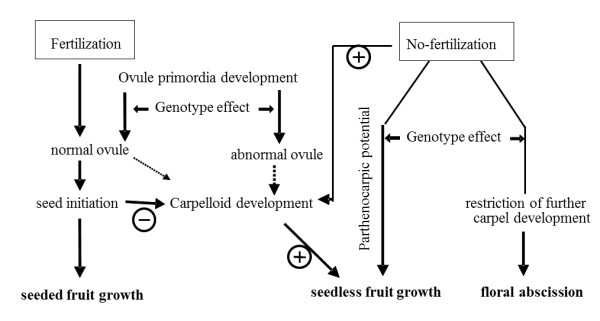
**The proposed model indicating the role of carpelloid structures (CLS) in parthenocarpic fruit development**. Genotypes have genetic potential for parthenocarpic fruit set, which becomes only expressed in the absence of fertilization. Abnormal ovules may convert into carpelloid structures; however, growth of carpelloid structures only becomes prominent in the absence of fertilization/seed initiation, as developing seeds suppress the growth of carpelloid structures. The carpelloid structures mimic the role of seeds and support parthenocarpic fruit growth. (solid lines represent our experimental findings and dashed lines represent likely routes).

Based on our findings we postulate a model indicating the role of fertility, aberrant ovules and carpelloid growth in parthenocarpic fruit set and development (Figure 6). Carpelloid development positively reinforces fruit growth, particularly in genotypes showing parthenocarpic potential. Abnormal ovules may convert into carpelloid structures, however, growth of carpelloid structures only becomes prominent in the absence of fertilization, indicating fertility as an important determinant of their development. In agreement with this model, genotype Line 3 showed reduced fertility and developed more carpelloid structures. Upon fertilization, normal seed development occurs, inducing fruit set but possibly suppressing carpelloid proliferation. Facultative parthenocarpy is widely present in *C. annuum *genotypes, and the absence of fertilization allows the parthenocarpic potential to be expressed, and at the same time induces carpelloid proliferation, possibly following the homeotic transformation of abnormal ovules.

## Methods

### Greenhouse conditions

The genotypes and greenhouse conditions used in different experiments are summarized in Additional file 4. In all the experiments, seeds were transferred on rockwool cubes with regular supply of nutrient solution [32]. Seedlings were transplanted on rockwool slabs at a density of 2.5 plants m^-2 ^in a compartment of a multispan Venlo-type glasshouse or in an air conditioned glasshouse, Wageningen, The Netherlands. Supplemental lighting by high pressure sodium lamps (Philips, SON-T, 600 W) for 16 hours (from 06.00 to 22.00) provided a minimum photon flux density of 125 μmol m^-2 ^s^-1 ^at the crop level. The terminal flower was removed from all plants at anthesis to support vegetative growth.

### Occurrence of parthenocarpy among *C. annuum *genotypes

*C. annuum *(Table, 1) genotypes were selected on the basis of their blocky appearance and seed number (Additional file 4: Exp 1). In total, 70-150 emasculations were performed in each genotype using 10 plants per genotype and fruit set was evaluated when fruits were ripe.

### Genotype effect on number and weight of carpelloid structures

Five genotypes: Parco, California Wonder 100 (CW), Riesen v. Californien (RVC), Bruinsma Wonder (BW), and Line 3 were arranged in one row of 8 plants at two temperatures (20/18°C, 16/14°C D/N) (Additional file 4: Exp 2). Two treatments (induced pollination or prevent-pollination) were completely randomized within the row. Induced pollination was done by vibrating the stem two times per week. Prevent-pollination was done by applying lanolin paste on the stigma of the flowers [33]. In genotypes Parco, RVC, CW and Line 3, flowers were given the treatments till three fruits per plant were obtained. In genotype BW, two flowers (one on main branch and one on a side branch) were treated at each of 20 nodes. Mature red fruit were harvested and their length, diameter and fruit fresh weights were recorded. Those seedless fruits that reached minimum of 50% of the weight of seeded fruit were considered as parthenocarpic and were used in our analysis while remaining were considered as knots. The number of seeds and number of carpelloid structures was counted in each fruit and each carpelloid structure was weighed.

### Ovule development in *C. annuum*

Line 3 and BW were inbred lines with high and medium potential to set parthenocarpic fruits [16] while Orlando (OR) was a fourth-generation inbred line developed from Orlando-F1 (De Ruiter seeds) (Additional file 4: Exp 3). Flowers were collected at 3-4 days before balloon stage. Pericarp was removed and morphological analysis of ovule development was conducted in the laboratory by using a field-emission cryo-scanning electron microscopy (SEM) (Jeol 6300F), equipped with an Oxford CT 1500HF cyro-stage system [34].

### Correlation of abnormal ovule development with reduced seed set and enhanced development of carpelloid structures

Two set of experiments were conducted (Additional file 4: Exp 4). In first experiment, genotypes Line 3 and OR were used to evaluate the occurrence of carpelloid structures. Flowers were tagged at 2 days before anthesis and allowed to pollinate naturally. Developing ovaries were harvested at 2 days of interval, dissected and evaluated for the presence of carpelloid structures by visual inspection. With the same set of genotypes, percentage of aberrant ovules and number of seeds was evaluated. Flowers (n = 6) were collected randomly at or around the anthesis stage. After removing the carpel, ovules were scraped smoothly in a water medium on a clean slide and the frequency of abnormal ovules was observed under optical microscope (Leitz Aristoplan). Seed set was counted when fruits reached the maturity (red) in both genotypes. In second experiment, genotypes Line 3 and BW were used. To evaluate the female fertility, plants were grown at day/night temperature of 14/16°C (low), 18.20°C (normal) and 22/24°C (high) and pollination was induced by vibrating the main stem two times per week. Number of seeds was counted when fruits reached the maturity (red) in both genotypes.

### Pollen viability and germination

Pollen grains of BW were collected from normal temperature (20/20°C day/night) and low night temperature (20/10°C day/night) in morning time (9.00-10.00 PM) (Additional file 4: Exp 5). To test the pollen viability, hydrated pollens were dissolved in FDA solution [35] and scored under fluorescence microscope. Pollen which fluoresced brightly under fluorescence was scored as viable. For pollen germination, the hanging drop technique was employed following published procedures [36] with some modifications. A liquid medium containing 0.25 mM MES (pH5.9), 15% (w/v) PEG 4000, 2% (w/v) sucrose, 700 ppm Ca (NO_3_)_2_, 100 ppm H_3_BO_3_, 200 ppm MgSO_4_, 100 pm KNO_3_) was used. Germination was considered only when germinating tube was larger or equal to the size of the pollen. Viability and germination percentages were determined, using 10-12 replicates of about 20-40 selected grains.

### Relation between parthenocarpy and carpelloid structures

Genotype BW with moderate potential for parthenocarpy was used in the experiment (Additional file 4: Exp 6). To obtain seeded fruits, flowers were tagged at anthesis and allowed to pollinate naturally. To obtained seedless fruit, flowers were emasculated two days before the expected date of anthesis and stigmas were cover with the lanolin paste or lanolin paste containing 0.05% 1-Naphthaleneacetic acid (NAA) or Gibberellic acid (GA_3_) [33]. Fifteen plants per treatment were used. On each plant, two flowers (one on main branch and one on a side branch) were treated at each of 20 nodes. All the fruits were harvested at mature red stage and their length, diameter and fruit fresh weights were recorded. Criteria to define parthenocarpic fruit and knot were the same as mentioned earlier (Exp.2). The number of seeds and number of carpelloids structures was counted in each fruit and each carpelloids structure was weighed.

### Inheritance of parthenocarpy and its relation with carpelloid structures

In order to understand the genetics of parthenocarpy and a possible association with carpelloids structures, genetics of both traits were evaluated in cross progenies of Line 3 (Additional file 4: Exp 7). Line 3 was used as a parthenocarpic parent (Pp) and Lamuyo B, ORF2#1, and Parco as non-parthenocarpic parents (Pn). F_2 _progenies were obtained for all three crosses, and also a backcross with Line 3 in the cross with Lamuyo B. The flowers (15-20) were emasculated prior to anthesis and tagged. All the fruits were harvested at the mature red stage. Length, diameter and fruit fresh weights were recorded for individual fruits. In each fruit, carpelloids structures were counted and mass were weighed. All three crossing population were evaluated and mono- or digenic-models were tested to understand the genetics behind parthenocarpy and carpelloids structures.

### Sequence analysis of *CaARF8*

Young leaf material from Line 3, BW and OR (Additional file 4: Exp 8) was collected for DNA extraction. Primers for PCR amplification were designed against pepper, tomato and potato *ARF8 *EST sequences available from the Sol Genomics Network (SGN), http://solgenomics.net/ (Additional file 5). SEFA PCR was used to amplify non-transcribed regions. PCR products were cleaned with the Invitek MSB^® ^Spin PCRapace. 120 ng of PCR product per reaction was sent with the appropriate sequencing primer (12 pmol) to ServiceXS, Leiden, The Netherlands. Resulting chromatograms were manually trimmed and checked for calling errors. Contigs were built by using Contig Express of the Invitrogen Vector NTI suite Version 10.4.

### Statistical analysis

Experiments and their statistical treatment are listed in additional file 4. For experiment 3 and 4, one way analysis of variance (ANOVA) was used, and treatment effects were tested at 5% probability level using F-test. For experiment 5, the effect of each treatment on each genotype at each temperature was tested separately by using a one way analysis of variance (ANOVA). Mean separation was done by student's t-test. Data processing and statistical tests were carried out with SPSS 15.0. The inheritance of parthenocarpy was tested by using chi square distribution with 1 degree of freedom at the 0.05 level of significance to test the null hypothesis that parthenocarpy was controlled by a single recessive gene. Carpelloid inheritance was tested using a chi square distribution, with different mono- or digenic models.

## Authors' contributions

AT performed the experiments, interpretated the data and drafted the manuscript with guidance from EH, RO, REV and AVS. AVS and MEJH cloned and sequenced the *CaARF8 *gene. AT, EH, RO and AVS revised the manuscript. LBX performed the selection of Line 3 and provided seeds for this work. All authors read and approved the final manuscript.

## Supplementary Material

Additional file 1 **Pollen viability and germination in *Capsicum annuum *genotypes**. Pollen viability and germination for genotypes Bruinsma Wonder and Lamuyo B grown at normal (20/20 °C) and low (20/10°C) day/night temperature. Different letters indicate significant differences between genotype-temperature combinations according to the LSD-test (***P ***= 0.05, ***n ***= 5-7 replicates). Click here for file

Additional file 2 **Fruit characteristics used in the segregation analysis**. A-B: Fruit shape and size of genotype Line 3, C-E: seedless fruit of shiny appearance and pointy bottom (C), and large depression on bottom (D), and small size (E); F- H: small size knots of partial dull appearance (F), big (G), and small (H) knots of fully dull appearance; I: seeded fruit. Plants were grown at 21/19°C D/N temperature. Scale bars: 1 cm (A-I). Click here for file

Additional file 3 ***Capsicum annuum ARF8 *genomic sequence in genotypes Line 3, Orlando and Bruinsma Wonder**. Exons are marked green, dark grey or light gray, depending on their correspondence to our cDNA clone, the Arabidopsis coding sequence or a Solgene EST, respectively, the translation start is marked yellow the miRNA167 binding site is marked blue. Click here for file

Additional file 4 **Summary of experimental setup (genotypes and greenhouse conditions) used in different experiments (Excel spreadsheet)**. The set of genotypes, their parthenocarpic fruit set potential, their origin, temperature set point and realized temperatures, cultivation method (one or two branch pattern system), and start month and end of the experiments. Click here for file

Additional file 5 **Primer sequences (Excel spreadsheet)**. Primer sequences used to amplify ***Capsicum annuum CaARF8 ***gene sequences. Click here for file
