# Supplementary figures and images for: Parthenocarpic potential in Capsicum annuum L. is enhanced by carpelloid structures and controlled by a single recessive gene
Source: BMC Plant Biol. 2011 Oct 21;11:143. doi: 10.1186/1471-2229-11-143 (PMC3214887; doi:10.1186/1471-2229-11-143)

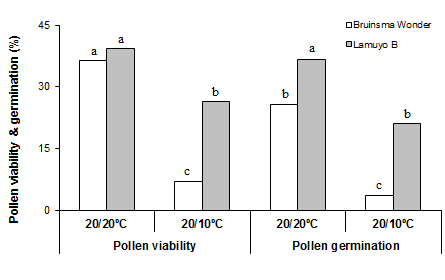

Supplement: Additional file 1 — Pollen viability and germination in Capsicum annuum genotypes. Pollen viability and germination for genotypes Bruinsma Wonder and Lamuyo B grown at normal (20/20 °C) and low (20/10°C) day/night temperature. Different letters indicate significant differences between genotype-temperature combinations according to the LSD-test (P = 0.05, n = 5-7 replicates). [file 1471-2229-11-143-S1.TIFF]

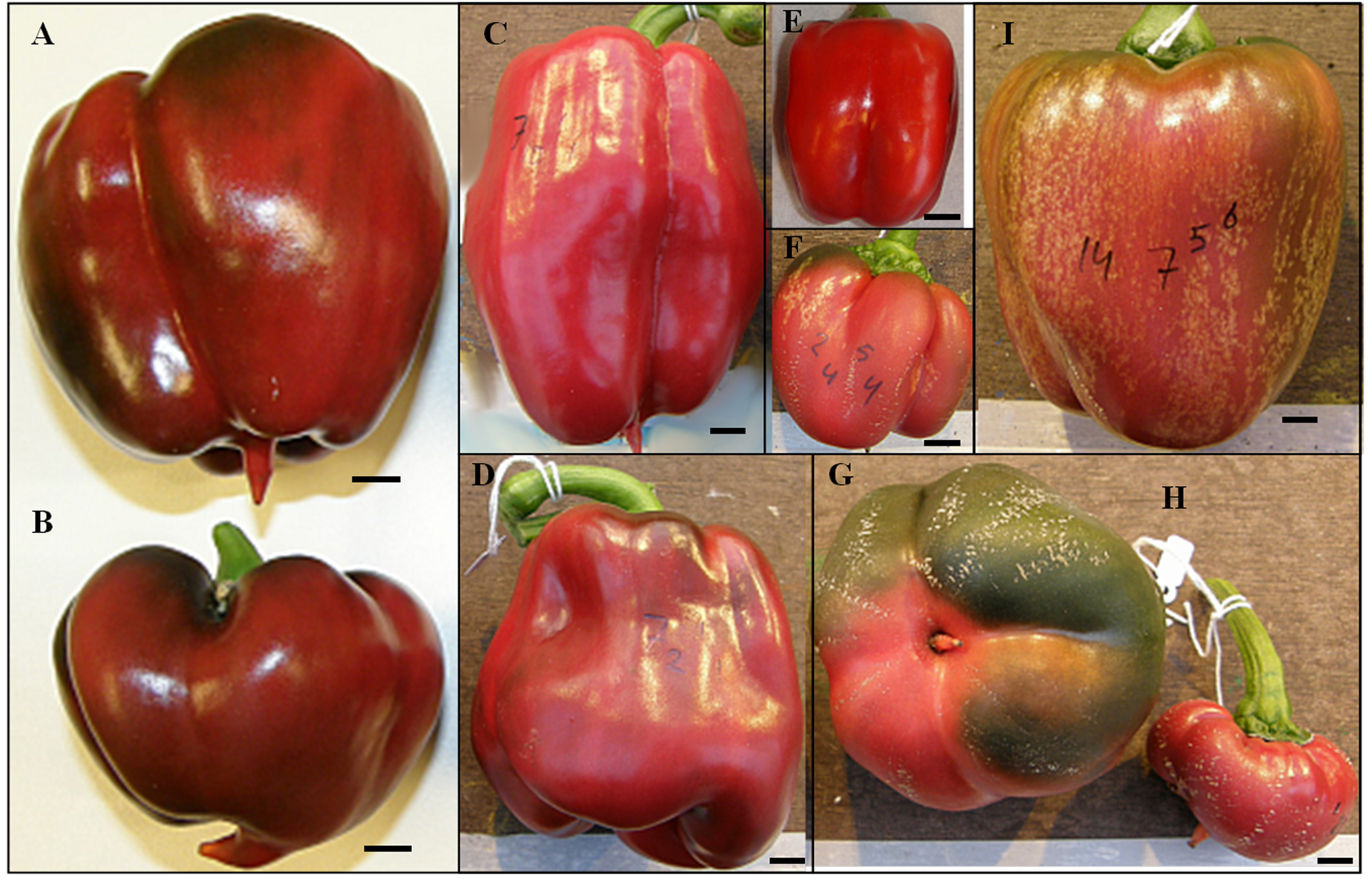

Supplement: Additional file 2 — Fruit characteristics used in the segregation analysis. A-B: Fruit shape and size of genotype Line 3, C-E: seedless fruit of shiny appearance and pointy bottom (C), and large depression on bottom (D), and small size (E); F- H: small size knots of partial dull appearance (F), big (G), and small (H) knots of fully dull appearance; I: seeded fruit. Plants were grown at 21/19°C D/N temperature. Scale bars: 1 cm (A-I). [file 1471-2229-11-143-S2.TIFF]

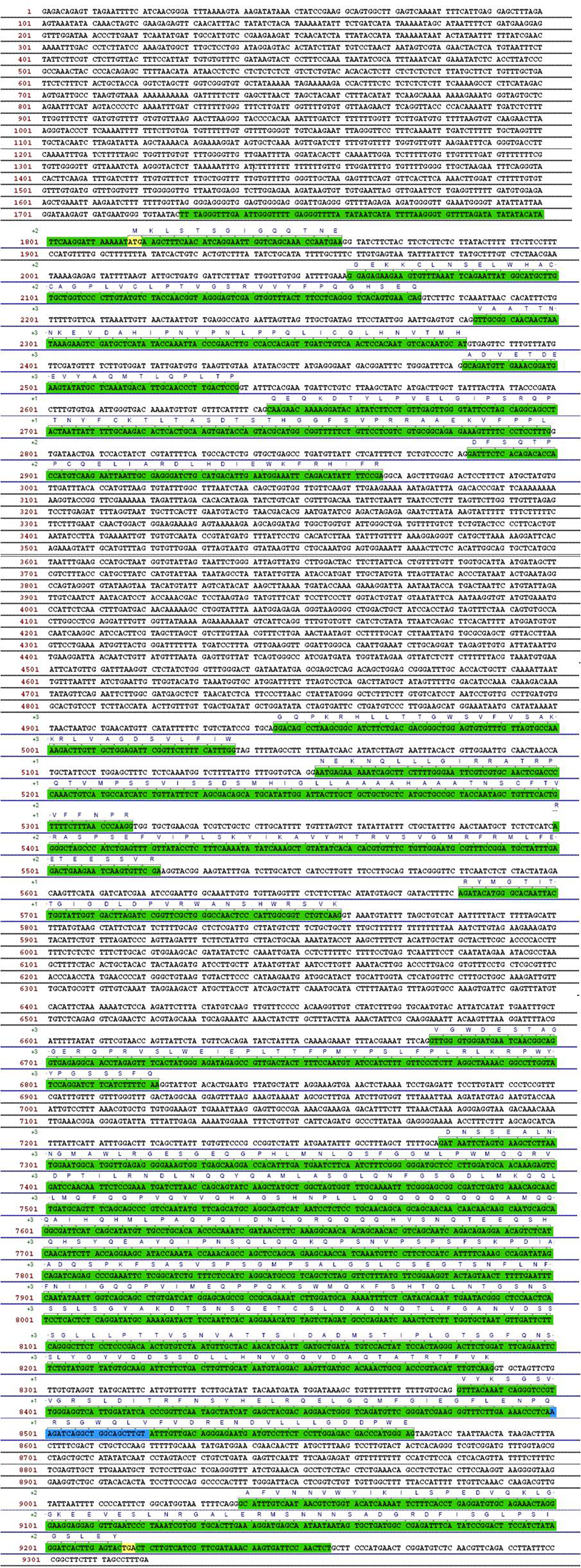

Supplement: Additional file 3 — Capsicum annuum ARF8 genomic sequence in genotypes Line 3, Orlando and Bruinsma Wonder. Exons are marked green, dark grey or light gray, depending on their correspondence to our cDNA clone, the Arabidopsis coding sequence or a Solgene EST, respectively, the translation start is marked yellow the miRNA167 binding site is marked blue. [file 1471-2229-11-143-S3.TIFF]
